# The Diagnostic Funnel: A Multi-Stage Diagnostic Selection Protocol for Minimally Invasive Sacroiliac Joint Fusion

**DOI:** 10.7759/cureus.116659

**Published:** 2026-09-21

**Authors:** Koleas Zumbro, Lawrence Maccree

**Affiliations:** 1 Neurosurgery, Lincoln Memorial University DeBusk College of Osteopathic Medicine, Harrogate, USA; 2 Neurosurgery, Methodist Medical Center, Oak Ridge, USA

**Keywords:** chronic low back pain (clbp), diagnostic funnel, intra-articular corticosteroid injection, minimally invasive sacroiliac joint fusion, minimally invasive surgery, patient selection criteria, provocative physical testing, sacroiliac joint fusion, sacroiliitis, treatment outcome

## Abstract

Background

Minimally invasive sacroiliac joint fusion (MI SIJF) is an established intervention for chronic sacroiliac joint dysfunction, yet outcome variability across the literature suggests that surgical success may heavily depend on preoperative selection accuracy. Traditional diagnostic pathways relying on transient anesthetic blocks are susceptible to false-positives and variable responder rates. The purpose of this study is to evaluate the clinical effectiveness of a rigorous, multi-stage selection protocol, termed the "diagnostic funnel," which utilizes a therapeutic durability threshold to ensure diagnostic specificity and optimize outcomes following MI SIJF.

Methodology

A retrospective chart review of 17 patients who underwent 19 MI SIJF procedures between January 2024 and August 2025 was conducted. To qualify for surgery, patients were required to meet all three stages of the “diagnostic funnel”: 1) chronic non-radicular pain (>6 months) with a positive Fortin finger sign, 2) at least three out of six positive provocative physical maneuvers (SI [sacroiliac] compression, ASIS [anterior superior iliac spine] distraction, Gaenslen’s test, FABER [flexion, abduction, external rotation)], thigh thrust, and Yeoman’s test), and 3) a "durability threshold" defined as at least 70% pain relief maintained for a minimum of 14 days following an image-guided intra-articular corticosteroid injection. Surgical success was measured at the six-month follow-up by mean percent reduction in visual analog scale (VAS) pain scores and the proportion of patients achieving the minimal clinically important difference (MCID, defined as a ≥20-point VAS reduction).

Results

The cohort demonstrated 100% adherence to the multi-stage selection protocol. The most frequently positive provocative maneuvers were Gaenslen’s test (94.7%), thigh thrust (89.5%), and FABER (89.5%). At six months postoperatively, patients achieved a mean VAS pain score reduction of 74.7%. Additionally, 94.4% of procedures successfully met or exceeded the MCID threshold for clinical success.

Conclusion

In this retrospective series, preoperative patient selection utilizing the multi-stage "diagnostic funnel" was associated with high clinical success rates. Replacing transient local anesthetic blocks with a 14-day corticosteroid "durability threshold" provides a more stable prognostic indicator of true intra-articular pathology. Adopting this diagnostic framework may allow spine surgeons to increase diagnostic specificity and consistently optimize postoperative pain reduction in MI SIJF patients.

## Introduction

Chronic low back pain remains one of the most challenging conditions to diagnose and treat, often leading to a diagnostic dilemma for spine surgeons and pain management specialists. While lumbar disc pathology and facet joint arthrosis are frequently suspected, clinical evidence suggests that the sacroiliac (SI) joint is the primary pain generator in approximately 15% to 30% of patients presenting with non-radicular low back pain [1,2]. However, the SI joint remains the great mimicker of the lumbar spine and hip, as its symptomatic profile frequently overlaps with lumbar disc herniation, facet arthropathy, and hip osteoarthritis [3]. While the efficacy of minimally invasive sacroiliac joint fusion (MI SIJF) has been well documented in recent years, the variability in reported responder rates across studies suggests that surgical success may depend heavily on the precision of the preoperative diagnostic workup.

In a previous report of the same cohort, we observed a mean pain reduction of 74.7% and a 94.4% success rate at six months [4]. The present study focuses on the detailed selection protocol used in that series and examines whether a multi-stage “diagnostic funnel” may contribute to these outcomes. The first stage of this funnel relies on a detailed clinical history and physical examination. Pathognomonic signs, such as the Fortin finger sign, in which a patient points directly to the posterior superior iliac spine, raise initial clinical suspicion [5]. This is followed by provocative physical testing, in which a cluster of at least three of six positive maneuvers has been shown to have high diagnostic sensitivity [6,7]. However, the final step in the diagnostic pathway - the intra-articular injection - lacks consensus on the threshold for surgical candidacy. Landmark multicenter trials, such as the INSITE [8] and iMIA [9] studies, utilized a 50% pain relief threshold from transient anesthetic blocks for inclusion. Yet, recent evidence suggests that short-acting anesthetic blocks may be susceptible to false-positives due to the placebo effect or anesthetic "leak" into the lumbosacral plexus [10]. To address these diagnostic inconsistencies, some clinicians have advocated for the use of therapeutic corticosteroid injections as a prognostic tool. Unlike the snapshot provided by local anesthetics, a response to corticosteroids requires a biological modulation of joint inflammation. Schneider et al. [11] suggest that a sustained response to anti-inflammatory modulation provides a more stable indicator of the joint as a chronic pain generator.

The primary objective of the present study is to evaluate the clinical effectiveness of a rigorous, multi-stage selection protocol - the "diagnostic funnel" - as a validation tool for surgical candidacy. The secondary objective is to describe the surgical outcomes achieved utilizing this standardized framework to identify patients most likely to benefit from MI SIJF. By strictly requiring a positive Fortin finger sign, a 3-test provocative cluster, and a sustained 14-day response to corticosteroids, we aim to determine if a refined selection protocol ensures the SI joint is the primary pain generator and optimizes long-term surgical success. We hypothesized that a stricter diagnostic threshold (sustained ≥70% pain relief for ≥14 days after corticosteroid injection) would be associated with favorable postoperative pain reduction following MI SIJF.

This article was previously presented as a meeting abstract and poster presentation at the Lincoln Memorial University DeBusk College of Osteopathic Medicine’s 10th Annual Essentials of Clinical Medicine CME Conference on June 12 and 13, 2026.

## Materials and methods

Study design and patient population

This study was a retrospective chart review of 17 patients who underwent MI SIJF between January 2024 and August 2025. Two of the participants underwent bilateral SI fusions at separate time points, resulting in a total of 19 MI SIJF procedures in the cohort. The study was conducted in accordance with the Declaration of Helsinki and received Institutional Review Board exemption from the Covenant Health Institutional Review Board. A waiver of informed consent was granted due to the study's retrospective design and the use of de- identified patient data.

The diagnostic funnel (inclusion criteria)

To be included in the study, patients were required to have completed a standardized, multi-stage "diagnostic funnel" and undergo an MI SIJF with our surgeon. This protocol mostly aligns with established clinical diagnostic criteria for SI joint dysfunction, including guidelines established by the International Society for the Advancement of Spine Surgery (ISASS) and the North American Spine Society (NASS) [12,13], as well as validated physical examination diagnostic clusters [6,7].

All patients progressed through a three-stage screening process to confirm the SI joint as the primary pain generator, as outlined in Table 1.

**Table 1:** The diagnostic funnel established to ensure patients were good candidates for SI joint fusion. ASIS, anterior superior iliac spine; FABER, flexion, abduction, external rotation; NSAIDs, nonsteroidal anti-inflammatory drugs; PT, physical therapy; ROM, range of motion; SI, sacroiliac; SLR, straight leg raise test

Diagnostic Phase	Clinical Metric	Inclusion Requirement
Clinical history	Duration of pain	>6 months (chronic)
	Physical limitations	Limitations in walking, sitting, standing, stair climbing, lifting, carrying, and/or straightening up
	Pain localization	Positive Fortin finger sign (pain at posterior superior iliac spine)
	Conservative care	Failure or intolerance to at least 6 weeks of PT/manual manipulation and/or NSAIDs
	Confirmatory imaging	CT or MRI of SI joints demonstrating spondyloarthropathy (sclerosis, degeneration)
Physical examination	Provocative maneuvers	3 out of 6 positive provocative maneuvers (SI compression, ASIS distraction, Gaenslen’s test, FABER, thigh thrust, and Yeoman’s test)
	Differential diagnosis	Negative exam findings for hip (ROM) and lumbar (SLR)
Diagnostic corticosteroid injections	Guidance	Image-guided (fluoroscopy or CT) w/ contrast
	Threshold	At least 70% pain relief for a minimum of 2 weeks after SI corticosteroid injection

Provocative maneuvers

The second stage of our “diagnostic funnel” required our patients to have at least three of six provocative maneuvers suggestive of sacroiliitis be positive. The provocative maneuvers that were performed on our patients are described below:

1. SI compression: with the patient in a lateral decubitus position, a downward vertical force is applied to the iliac crest. This maneuver compresses the joint surfaces to elicit a pain response.

2. ASIS distraction: with the patient supine, a posterolateral force is applied to both anterior superior iliac spines (ASIS). A positive test reproduces the patient's familiar SI joint pain.

3. Gaenslen’s test: the patient lies near the edge of the table, with one leg flexed toward the chest and the other extended off the table. This creates a torsion force across the pelvic ring.

4. FABER (flexion, abduction, external rotation): with the patient supine, the foot of the involved side is placed on the opposite knee. A downward force is then applied to the medial aspect of the flexed knee while stabilizing the opposite iliac crest. This maneuver (also known as Patrick’s test) places the SI joint in a position of combined stress to elicit a localized pain response.

5. Thigh thrust: the patient is supine with the hip and knee flexed to 90 degrees. The surgeon applies downward pressure along the long axis of the femur, thereby providing a posterior shear force to the SI joint.

6. Yeoman’s test: with the patient in a prone position, the examiner stabilizes the involved-side SI joint with one hand while extending the patient's hip with the other, keeping the knee flexed at 90 degrees. This creates a longitudinal stretch on the anterior SI joint ligaments and serves as a highly specific maneuver for identifying anterior SI joint irritability.

To eliminate inter-rater variability and ensure diagnostic consistency, all provocative maneuvers were performed and clinically verified by the same single operating surgeon during the preoperative evaluation.

Surgical technique

All procedures were performed by a single surgeon using a lateral percutaneous minimally invasive approach. Patients were positioned in either the prone or lateral decubitus position under general anesthesia. Under fluoroscopic guidance utilizing lateral, inlet, and outlet views, lateral transiliac access was obtained. In each case, three hydroxyapatite-coated, self-drilling, and self-harvesting titanium screws (8.7 mm diameter; lengths ranging from 35 mm to 50 mm) were utilized for stabilization. The implants were advanced along a trajectory perpendicular to the SI joint. Criteria for optimal implant placement included careful visualization of the neural foramina, assessment of the anteroposterior (AP) diameter of the sacrum, and ensuring that all hardware remained strictly below the alar line. Beyond the passage of the self-harvesting screws, which facilitated the collection of local autograft, no specific joint decortication was performed, and no additional allograft was used.

Postoperative protocol and perioperative management

Following the procedure, a standardized postoperative protocol was implemented for all patients. To minimize the risk of surgical site infection, patients were prescribed a 10-day course of oral cephalexin 500 mg to be taken three times daily. Postoperative pain management was standardized with a three-day course of hydrocodone/acetaminophen (10 mg/325 mg) for acute analgesic control. Regarding mobility, patients were restricted to toe-touch weight-bearing for the first two postoperative weeks, during which the use of assistive devices, such as a walker or crutches, was permitted to maintain stability while ensuring no significant load was placed on the operative limb. After the initial two-week period, patients began a gradual weaning process from assistive devices, transitioning toward weight-bearing as tolerated based on clinical progression.

Outcome measures and statistical analysis

The primary outcome measured was the change in visual analog scale (VAS) pain scores (utilizing a standard 0-100 scale) from preoperative baseline to six months postoperatively. VAS scores were collected in the clinic via written patient self-reports at each visit and subsequently confirmed by the surgeon during the clinical encounter. Surgical success was defined as achieving the minimal clinically important difference (MCID), set a priori as a ≥20-point reduction in VAS pain score from baseline to six months, in accordance with the established threshold utilized in the iMIA trial [9]. For the final analysis, the mean percent pain reduction and the overall percentage of patients meeting the MCID criteria were calculated. One procedure lacked a complete six-month VAS score and was excluded from the MCID analysis (n=18).

## Results

Patient demographics

A total of 19 MI SIJFs were performed across 17 patients. Table 2 provides the demographic characteristics of all the patients in our cohort. For the two patients who underwent staged bilateral procedures, each surgery was treated as an independent observation for the purpose of clinical and statistical analysis. All patients included in the analysis had a confirmed diagnosis of SI joint dysfunction, underwent MI SIJF, and completed 10-day, six-week, 10-week, and six-month follow-up visits.

**Table 2:** Patient demographics of our cohort. *Calculated based on 19 independent surgical observations.

Characteristic	Value (N = 19*)
Age (years)	
Mean (± SD)	65.5 ± 10.1
Median (range)	65 (47-82)
Gender, n (%)	
Male	10 (52.6%)
Female	9 (47.4%)
Diabetes status, n (%)	
Diabetics	10 (52.6%)
Non-diabetics	9 (47.4%)
BMI, n (%)	
Underweight (<18.5)	0 (0.0%)
Healthy weight (18.5-24.9)	0 (0.0%)
Overweight (25.0-29.9)	6 (31.6%)
Obese (≥30.0)	13 (68.4%)
Osteoporosis status, n (%)	
Diagnosed with osteoporosis	1 (5.3%)
No known osteoporosis	18 (94.7%)
Smoking status, n (%)	
Current or former smoker	13 (68.4%)
No smoking history	6 (31.6%)

Cohort diagnostic validation

The retrospective chart review of our 19 MI SIJFs confirmed 100% adherence (n=19/19) to the multi-stage diagnostic protocol required for surgical candidacy. Each patient underwent the full "diagnostic funnel," as outlined in Table 1, ensuring that the SI joint was the primary pain generator before fusion.

Clinical History

Review of our cohort’s initial history and physical documentation revealed that all patients met the clinical history requirements outlined in Table 1. Specifically, all of our patients had chronic SI pain (>6 months of symptoms), multiple physical limitations due to their SI joint pain, a positive Fortin finger sign, and failure or intolerance to at least six weeks of physical therapy, manual manipulation (chiropractic or osteopathic manipulation), and/or NSAIDs. In addition, all of our patients had either a CT or an MRI of the SI joints demonstrating spondyloarthropathy (sclerosis, degeneration).

Physical Examination and Provocative Testing

Preoperative physical examination records demonstrated that all of our patients (n=19/19, 100%) met the 3-out-of-6 threshold for provocative maneuvers suggestive of SI joint pathology. As listed in Table 3, the frequency of positive responses for specific provocative maneuvers was highest for Gaenslen’s test (n=18/19, 94.7%), thigh thrust (n=17/19, 89.5%), and FABER (n=17/19, 89.5%) tests, followed by Yeoman’s test (n=9/19, 47.4%), SI compression (n=7/19, 36.8%), and ASIS distraction (n=5/19, 26.3%). Both hip and lumbar pathologies were also ruled out for all of our patients, as documented in the initial history and physical documentation.

**Table 3:** Preoperative provocative physical testing results. FABER, flexion, abduction, external rotation; SI, sacroiliac; ASIS, anterior superior iliac spine

Provocative Maneuver	Clinical Testing Mechanics	Positive Results (n/N)	Positive Percentage (%)
Gaenslen’s test	Torsion force across pelvic ring via simultaneous leg flexion/extension	18 / 19	94.70%
Thigh thrust	Posterior shear force applied along femur axis	17 / 19	89.50%
FABER (Patrick’s test)	Combined flexion, abduction, and external rotation stress	17 / 19	89.50%
Yeoman’s test	Prone hip extension creating anterior ligament stretch	9 / 19	47.40%
SI compression	Vertical downward pressure on iliac crest in lateral decubitus	7 / 19	36.80%
ASIS distraction	Posterolateral distraction force applied to anterior superior iliac spines	5 / 19	26.30%

Diagnostic Injection Performance

All patients in our cohort underwent at least one image-guided SI corticosteroid injection that met the 70% relief for the two-week threshold. This high response to corticosteroid injections suggests that the pre-injection diagnostic workup successfully identified patients with intra-articular pathology before the injections were even administered. In addition, the 14-day sustained relief served as a clinical simulation for the surgical outcome.

Correlation with surgical outcomes

Strict adherence to this diagnostic protocol was correlated with the superior clinical outcomes reported in our previous analysis [4]. Specifically, the cohort achieved a 74.7% mean reduction in VAS pain scores at six months. Postoperative VAS score improvements across the cohort are illustrated in Figure 1.

**Figure 1:**
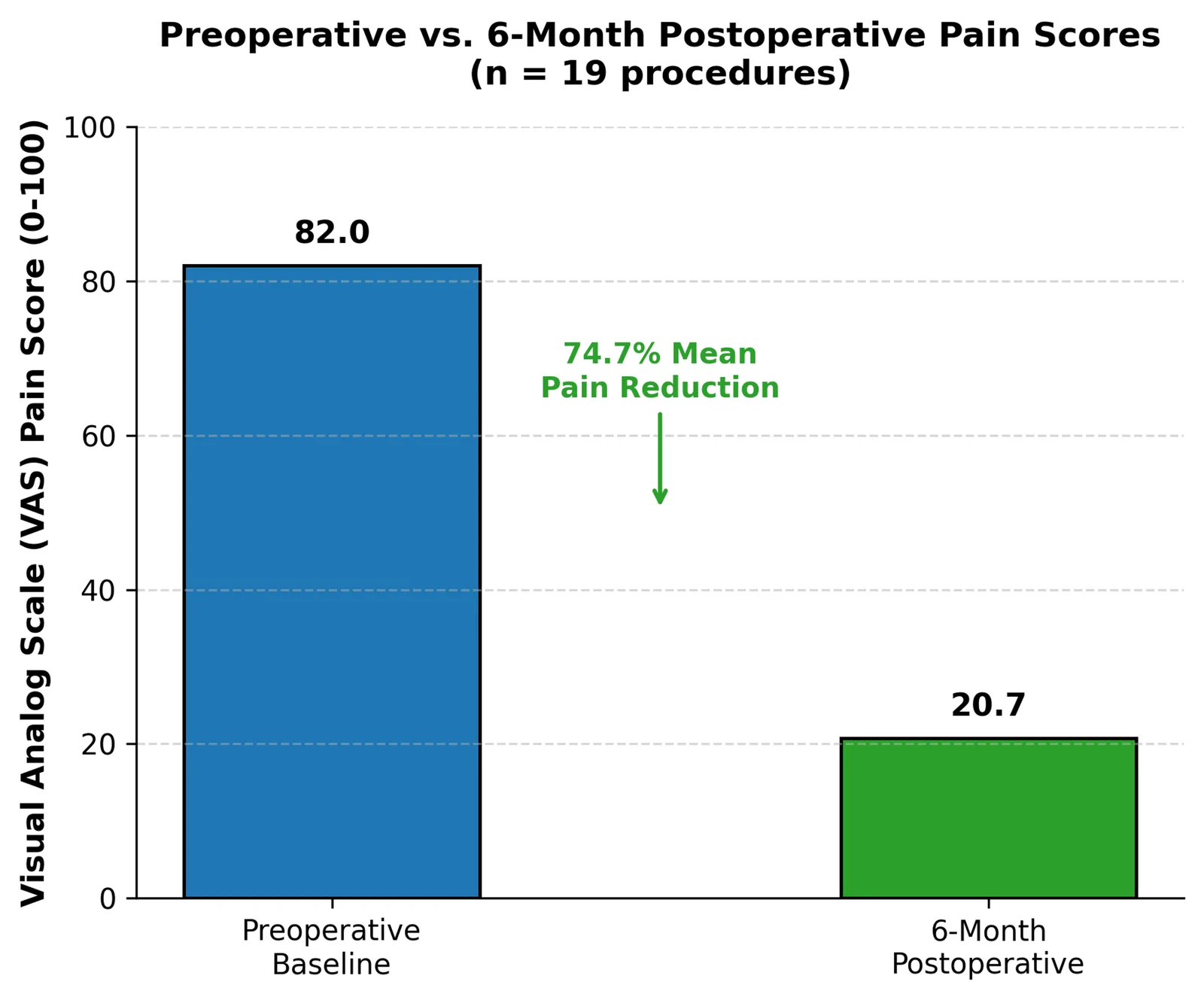
Preoperative versus six-month postoperative pain scores.

Furthermore, the 94.4% success rate for reaching the MCID suggests that the 70% relief for the two-week threshold is a reliable predictor of postoperative success in MI SIJF. Table 4 displays the specific breakdown of the surgical and clinical outcomes in our cohort.

**Table 4:** Summary of surgical and clinical outcomes at six months. MCID, minimal clinically important difference; VAS, visual analog scale

Outcome Parameter	Value / Measurement
Total surgical procedures (N)	19
Protocol adherence rate (n/N, %)	19/19; (100%)
Mean postoperative pain reduction (%)	74.70%
Proportion achieving MCID (≥20-point VAS reduction)	17/18; (94.4%)
Corticosteroid injection durability threshold	≥70% pain relief for ≥14 days

## Discussion

The primary objective of this study was to evaluate whether a rigorous, multi-stage diagnostic workup - the "diagnostic funnel" - predicts superior clinical outcomes following MI SIJF. Our findings demonstrate that our cohort that achieved a mean pain reduction of 74.7% strictly adhered to the protocol, which required at least three positive provocative tests and at least 70% relief for two weeks following an image-guided SI injection. While the efficacy of MI SIJF is well established, the variability in responder rates across the literature, ranging from 78.8% in the iMIA trial [9] to 94.4% in the present study, highlights the critical role of the "diagnostic funnel." Our findings are consistent with the hypothesis that rigorous patient selection may contribute to favorable outcomes, although the retrospective design and small sample size prevent isolation of the selection protocol as the primary causal factor.

Anesthetic snapshot versus therapeutic durability

The primary limitation of a standard diagnostic block (lidocaine or bupivacaine) is its transient nature. A 50% reduction in pain for two hours can be attributed to the placebo effect, patient expectation, or anesthetic tracking into the epidural space or the lumbosacral plexus [14]. In contrast, a corticosteroid injection requires a biological response - the reduction of synovitis and capsular inflammation - which typically manifests 48 to 72 hours post-injection and lasts for several weeks. While most SI joint literature, including the INSITE [8] and iMIA trials [9], relies on a relief threshold greater than 50% from a transient anesthetic block, our protocol used a 14-day corticosteroid response threshold. This approach is supported by the work of Schneider et al. [11], which suggests that a sustained response to anti-inflammatory modulation provides a more stable prognostic indicator of surgical success than the snapshot provided by local anesthetics alone.

By enforcing a 14-day response threshold, we ensured that the relief was not due to local anesthetic spread to adjacent nerve roots, which would have dissipated within hours. This "durability threshold" confirms that the pathology is truly intra-articular. Our 94.4% surgical success rate demonstrates that patients who respond to anti-inflammatory modulation over a sustained period are the ideal candidates for the permanent mechanical stabilization provided by an MI SIJF.

Mechanical stabilization as the logic for fusion

The strong correlation between the 14-day steroid response and the 74.7% mean surgical pain reduction provides a clear biomechanical narrative. The SI joint can exhibit clinically relevant mobility that is not captured by static morphological assumptions [15]. Provocative maneuvers such as Gaenslen’s test, Thigh Thrust, and FABER - which were positive in 94.7%, 89.5%, and 89.5% of our cohort, respectively - induce pain by creating shear and tension across an inflamed joint. If a corticosteroid injection can alleviate this pain for two weeks by chemically dampening the inflammation, then MI SIJF provides the logical mechanical solution: by rigidly stabilizing the joint, we eliminate the micro-motion and friction that cause the inflammation to recur.

Clinical implications for selection

We propose that requiring a minimum of 70% pain relief maintained over a 14-day window provides a higher degree of diagnostic specificity than the traditional 50% threshold from a transient anesthetic block utilized in the INSITE [8] and iMIA trials [9].

Adopting this stricter "diagnostic funnel" involves a clinical trade-off. In an outpatient spine practice, enforcing a 14-day therapeutic durability requirement will inevitably result in a higher "screen-fail" rate during initial evaluation, disqualifying patients who experience only transient or equivocal relief from local anesthetics. However, this upfront selectivity acts as a crucial protective filter. While high-volume, low-selectivity screening paradigms may increase immediate surgical conversion rates, they run a higher risk of operating on non-responder pathologies, leading to persistent postoperative pain, revision interventions, and unnecessary healthcare expenditure.

By intentionally prioritizing diagnostic specificity over surgical volume, the "diagnostic funnel" optimizes resource utilization for both patients and healthcare systems. Restricting surgery to candidates who demonstrate sustained anti-inflammatory responsiveness ensures that permanent mechanical stabilization directly targets an established, chronic intra-articular pain generator. For spine surgeons seeking to match the high responder rates presented in this series, shifting the diagnostic emphasis from a transient anesthetic snapshot to a verified window of therapeutic durability represents a more predictable approach to candidate selection.

Limitations

Several limitations must be acknowledged. First, the sample size of 19 MI SIJFs is relatively small compared to those of large multicenter studies. Second, the retrospective nature of the study introduces potential selection bias. Because the data were collected from a single-center, single-surgeon experience, the outcomes may reflect localized patient demographics or specific technical proficiencies that may not completely generalize across all surgical practices. Third, while the six-month follow-up period was sufficient to capture short-term success and MCID thresholds, it does not account for the long-term (multi-year) durability of the fusion or the potential development of adjacent segment disease. Additionally, because patients often received their diagnostic injections from various referring pain management specialists, the specific pharmacological details, including the exact corticosteroid agent, dose, and volume, were not available for extraction, preventing strict standardization. Finally, the lack of a comparative control group makes it difficult to definitively isolate the "diagnostic funnel" as the sole variable driving surgical success.

Furthermore, our cohort demonstrated a high prevalence of comorbidities known to impair surgical outcomes, including obesity, diabetes, and a history of smoking. Given the small sample size, we were unable to perform a multivariate analysis to control for these confounding variables in the present study, though this remains an active area of future investigation by our team. Another limitation is our reliance on the VAS as the sole outcome metric. While this paper focuses strictly on clinical pain outcomes associated with the selection protocol, it does not detail long-term radiographic osseointegration, though early postoperative imaging confirming implant placement and fusion status was previously documented in our initial cohort report [4].

Notwithstanding these limitations, a primary strength of this study lies in the clinical rigor of the multi-stage "diagnostic funnel" protocol. By establishing a strict diagnostic requirement - specifically demanding a 70% pain reduction sustained over a 14-day durability threshold - this framework intentionally prioritized high diagnostic specificity to ensure precise patient selection. The high postoperative responder rates observed demonstrate the clinical utility of this stringent strategy. Furthermore, while brief, the six-month follow-up period provided a reliable window to capture early recovery dynamics, including the transition through the 10-day "rapid relief window" and the successful achievement of the MCID. Future prospective, randomized trials comparing the standard 50% anesthetic threshold against this stricter 70% corticosteroid threshold are warranted to further validate these findings and standardize preoperative protocols.

## Conclusions

The success of MI SIJF is not merely a matter of surgical execution but dependent on meticulous preoperative patient selection. Our study demonstrates that by implementing a multi-stage "diagnostic funnel," comprising the Fortin finger sign, a 3-test provocative cluster, and a sustained 70% therapeutic response for 14 days, surgeons can achieve outcomes that exceed current industry benchmarks.

In this small retrospective series, a multi-stage selection protocol that included a sustained 70% response to corticosteroid injection for ≥14 days was associated with high rates of clinical success at six months. The 14-day corticosteroid window serves as a critical "clinical simulation," ensuring that the joint is not only the primary pain generator but is also responsive to the anti-inflammatory environment that mechanical stabilization aims to provide. As MI SIJF continues to evolve, these findings support further prospective evaluation of the "durability threshold" as a potential refinement of current selection criteria for MI SIJF.
